# Bifidobacterium- and Escherichia-dominant ecological guilds shape altered microbial metabolic capacity of the gut microbiome in tuberculosis patients

**DOI:** 10.3389/fcimb.2026.1751447

**Published:** 2026-07-21

**Authors:** Milyausha Yunusbaeva, Dina Sabirova, Liliya Borodina, Karina Khasanova, Aliya Yunusbayeva, Radick Altinbaev, Ildar Ganiev, Bayazit Yunusbayev

**Affiliations:** 1 Department of Molecular Biomedicine, Russian Federation Healthcare Ministry, Saint-Petersburg State Phthisiopulmonology Research Institute, Saint Petersburg, Russia; 2 Department of Tuberculosis Monitoring, Republican Clinical Antituberculous Dispensary, Ufa, Russia; 3 Institute of Fundamental Training and Technological Innovations, State University of Aerospace Instrumentation, Saint Petersburg, Russia; 4 Laboratory of Neurophysiology of Learning, Institute of Higher Nervous Activity and Neurophysiology, Russian Academy of Sciences, Moscow, Russia; 5 Department of Genetics and Biotechnology, Saint Petersburg State University, Saint Petersburg, Russia

**Keywords:** anaerobic glycolysis, bacterial guild, dysbioses, enterosignature, gut microbiome, homolactic fermentation, tuberculosis

## Abstract

**Introduction:**

The gut microbial community plays a key role in maintaining the host immune homeostasis. However, current analytical approaches analyze individual taxa rather than gut communities, thereby missing community-level functions performed by units, such as ecological guilds. Delineating ecological units is a promising approach for summarizing the functional output of microbes and their impact on the host.

**Methods:**

In this study, we investigated gut bacteria in 33 tuberculosis patients and 47 healthy controls using enterosignatures (ESs), ecological units of co-occurring bacteria related by function. We focused on detecting enterosignatures enriched in the gut communities of tuberculosis (TB) patients. For each patient-enriched enterosignature, we counted the metabolic pathways encoded by its member species. In this manner, we characterized the functional potential of ecological guilds enriched in TB patients. Finally, we tested whether ecological guilds correlate more closely with disease and host biomarkers.

**Results and Discussion:**

We show that inferred ESs represent reproducible units that facilitate proper comparison of identified ecological guilds to those observed in worldwide donor populations. Namely, dominant enterosignatures in the analyzed healthy donors reproduced the same ecological guilds observed among healthy individuals worldwide. In contrast, most TB patients carried two enterosignatures (ES-Bifi and ES-Esch) that were hallmarks of disturbed gut communities and atypical for healthy adults. We estimated the abundance of metabolic pathways encoded by member species of these patient-enriched ESs. We found that an increase in bacterial species comprising ES-Bifi and ES-Esch harbor an increased number of pathways for fermenting simple sugars, with end products such as acetate and lactate. A greater number of ecological guilds that ferment glucose to lactate might indicate an altered gut environment in patients, including increased acidity and disturbed carbohydrate flux. Taken together, our analyses suggest that ESs represent a biologically meaningful unit for reducing the complexity of the human gut microbiome and a tool for recognizing sharper patterns behind noisy taxonomic and functional diversity.

## 1 Introduction

Tuberculosis (TB) remains the deadliest infectious disease, imposing a significant global burden, especially in developing countries. In 2023, 8.2 million people were newly diagnosed with tuberculosis worldwide, an increase from 7.5 million in 2022 and 7.1 million in 2019 (World Health Organization, 2024). One contributing factor to this rise is variable treatment response, underscoring the urgent need to understand the host factors that influence differential host resistance and tolerance.

The gut microbiota plays a crucial role in maintaining host immune homeostasis not only in the intestine, but also systemically and can affect the mucosal immunity in the respiratory tract (Budden et al., 2017; Campbell et al., 2023; Libertucci and Young, 2019; X. V. Li et al., 2019). For instance, murine models of tuberculosis have shown that alterations in the gut microbiota can increase host susceptibility to *Mycobacterium tuberculosis* (MTB) infection (Khan et al., 2016). In addition, changes in the gut microbiota impair isoniazid-induced MTB clearance by reducing innate immunity and attenuating the CD4+ T cell response to MTB (Khan et al., 2016). On the other hand, there is evidence for a reciprocal relationship, as experimentally induced lung infection with *M. tuberculosis* can disturb the gut microbiota in mouse models of TB (Winglee et al., 2014). The precise mechanisms of this reciprocal gut-lung axis are not fully understood. It is known that during host inflammation, there is elevated trafficking of immune cells and inflammatory cytokines to the intestine, with effects on the local mucosal immunity. In the meantime, the commonly observed effect of host inflammation is depletion of obligate anaerobes in the gut and changes in the microbial metabolic output (de Gaay Fortman et al., 2026). However, host inflammation also disrupts mucosal hypoxia and increases intestinal lumen oxygen levels, thereby disturbing the anaerobic homeostatic community (Lee et al., 2024; Litvak et al., 2018).

Numerous human case-control studies have reported alterations in the taxonomic composition of the fecal community in TB patients (Hu et al., 2019; W. Li et al., 2019, 2024; Luo et al., 2017; Wang et al., 2022; Ye et al., 2022; Yu et al., 2023). However, the causal relationships between these taxonomic changes and lung infection remain poorly understood, in part due to variability in taxonomic associations and functional redundancy among bacterial species (Burke et al., 2011). In the human gut, like in any ecological niche (Louca et al., 2016), the environment filters and assembles bacterial species into communities based on function. As a result, bacterial species coexist with their ecological partners to serve ecological functions and form the so-called ecological guilds (Lee et al., 2024). Therefore, to understand their functional output and, if any, their impact on the host, it is essential to reconstruct co-occurring ecological partners and identify their joint metabolic output (Wu et al., 2021).

Co-occurring microbial species forming ecological guilds can be reconstructed as enterosignatures—a new concept—that, unlike discrete enterotypes (Arumugam et al., 2011), better represent continuous combinations of functional groups of bacterial species in the human gut. Enterosignatures (ESs) can be inferred using non-negative matrix factorization (NMF), a dimensionality reduction technique that used to model the hidden structure of the complex gut microbiome in terms of microbial groups (of co-occurring taxa) and their continuous mixtures (Frioux et al., 2023; Jiang et al., 2012; Raguideau et al., 2016). A previous study by Frioux et al. (2023) demonstrated that the human gut microbiome comprises at least five enterosignatures, each representing a stable, reproducible biological unit. Importantly, these enterosignatures and their combinations can capture the vast diversity of human gut microbiomes observed in Western and non-Western populations. In addition, ESs can describe both typical healthy gut microbiomes called “primary ESs”, and atypical microbiomes associated, for example, with antibiotic use, adverse host health conditions, and unstable infant fecal microbiomes.

In this study, we used the five enterosignature model, pre-computed using an NMF approach based on a global collection of 5,230 fecal metagenomes (Frioux et al., 2023). We opted for this pre-computed model because it captures the global variation in fecal metagenomes across 13 countries and allows projection of small samples. This model is sufficient to describe typical adult fecal microbiomes and various deviations in terms of atypical microbiomes (Frioux et al., 2023). We projected fecal microbiomes of TB patients and healthy donors to this pre-computed model and inferred globally observed enterosignatures in the analyzed case-control cohort: ES-Bact (primarily characterized by the genera *Bacteroides* and *Phocaeicola*), ES-Bifi (*Bifidobacterium* and *Streptococcus*), ES-Prev (*Prevotella*), ES-Esch (*Escherichia* and *Citrobacter*), and ES-Firm (genera within the phylum *Firmicutes*) (Frioux et al., 2023). Notably, we identified two atypical enterosignatures (ES-Bifi and ES-Esch) that were enriched in the fecal microbiomes of TB patients. Given that ESs may represent ecological guilds responding to gut environments, we hypothesized that an increase in atypical ESs might indicate alterations in the intestinal environments of TB patients (e.g., nutrient flux, oxygen concentration, and pH). To explore this hypothesis, we analyzed the differentially enriched metabolic pathways in subsets of patients with atypical enterosignatures, ES-Bifi and ES-Esch. Since patients were heterogeneous with respect to ESs, we reasoned that selecting a subset of patients with similar enterosignatures (ES-Bifi or ES-Esch) would enrich for metabolic pathways associated with these atypical ESs. Finally, we compared these ES-based inferences with results from conventional approaches that treat gut communities as collections of microbial taxa. For instance, we detected an unusual prevalence of fermentation pathways from simple sugars in atypical ESs that were not readily recognizable by conventional approaches. Moreover, atypical ESs showed stronger correlations with host biomarkers, such as impaired glucose metabolism in patients.

## 2 Methods

### 2.1 Study design, samples, and quality control

In this case-control study, we collected 80 fresh-frozen fecal samples from 47 healthy adult donors and 33 treatment-naive patients with active pulmonary tuberculosis admitted to the Republican Clinical TB Dispensary in Ufa, Russia, between 2019 and 2021. The metagenomic sequences from 47 healthy controls and a subset of TB patients were previously published in a previous study from our team (Yunusbaeva et al., 2024). All patients had newly diagnosed active pulmonary TB as confirmed by chest radiography and a positive sputum smear or were positive for *M. tuberculosis* based on the GeneXpert MTB/RIF test without evidence of rifampin resistance. Exclusion criteria included severe comorbidities, hemoptysis, hypoxia, extrapulmonary tuberculosis, a history of tuberculosis treatment, use of anti-TB drugs within the past 30 days, pregnancy or breastfeeding, and HIV infection. Fecal samples were collected from TB patients before treatment commenced. Control participants (n = 47) were recruited from staff members at the Institute of Biochemistry and Genetics of the Russian Academy of Sciences (Ufa, Russia) and matched to cases by age and sex. These control subjects had no unexplained symptoms or other conditions, including infectious, autoimmune, or gastrointestinal disorders. All subjects showed no radiographic abnormalities. Additional characteristics that may affect study results, including body mass index, smoking, lifestyle, and antibiotic usage, were not available. Before entering the study, all participants provided written informed consent.

Fresh stool samples from the participants were collected using sterile containers and immediately stored at -80 °C for further analysis. Bulk DNA was extracted from each donor’s fecal samples using the QIAamp DNA Stool Mini Kit (Qiagen, Netherlands). Sequencing libraries were prepared using the TruSeq DNA PCR-Free Sample Preparation Kit (Illumina, USA) according to the manufacturer’s instructions. Deep metagenomic sequencing was performed on the NovaSeq 6000 platform using 150-bp paired-end reads, yielding approximately 17 million reads per donor.

### 2.2 Raw sequence QC and preprocessing

We assessed sequence quality before and after trimming and decontamination using FastQC v0.11.9 (Wingett and Andrews, 2018). Trimmomatic v0.33 (Bolger et al., 2014) was employed to remove low-quality bases (Q20), and fastp v0.23.2 was used to clip Illumina adapters (S. Chen et al., 2018). Additionally, tandem repeats were removed using Tandem Repeats Finder v4.09 (Benson, 1999). Finally, the KneadData v0.10.0 pipeline from the bioBakery toolset was utilized to decontaminate reads originating from the human genome, transcriptome, and microbial RNA (McIver et al., 2018). Specifically, we used the human reference genome (hg37), the human reference transcriptome (hg38), and the SILVA release 128 ribosomal RNA reference databases. All the downstream analyses use shotgun metagenomic reads.

### 2.3 Enterosignature inference

To predict enterosignatures in the analyzed fecal microbiomes, we used publicly available five enterosignature model (https://enterosignatures.quadram.ac.uk/). Specifically, we inferred enterosignatures in the analyzed fecal microbiomes by projecting genus-level abundances (matrices) onto the pre-computed five-enterosignature model. The five enterosignature model was computed on a global collection of 5,230 fecal metagenomes (Frioux et al., 2023) representing donors from 13 countries in the Gut Microbiome Reference (GMR) panel (Hildebrand et al., 2021). We chose to use the pre-computed model because it captures the global diversity of typical (healthy) fecal microbiomes and is informative for distinguishing diverse alterations in atypical enterosignatures. Second, the underlying NMF approach enables the projection of small samples onto a model derived from large cohorts, greatly benefiting small-scale studies. According to the five enterosignature model, combinations of five distinct microbial guilds, ES-Bact, ES-Bifi, ES-Esch, ES-Prev, and ES-Firm are sufficient to represent diverse fecal metagenomes observed globally. Each enterosignature represents a microbial guild consisting of a unique set of co-occurring species that are likely serving an ecological function in the given gut environment (Frioux et al., 2023). For each analyzed donor (TB patient and healthy control), we estimated the relative proportion of enterosignatures in their fecal microbiome that sum up to 1.0. The quality of enterosignature prediction was assessed using the cosine similarity measure between the original genus-level abundance (matrix) and its enterosignature representation. We then visualized predicted enterosignature proportions using stacked bar plots, grouping fecal donors by case-control status and ordering within groups by the proportion of the prevalent enterosignature. Permutational multivariate analysis of variance (PERMANOVA) based on Aitchison distance was used to test overall compositional differences between groups. Statistical differences in enterosignature abundances between treatment-naive TB patients and healthy controls were tested using Wilcoxon rank-sum tests, by applying Benjamini-Hochberg correction for multiple comparisons. Two enterosignatures (ES-Esch and ES-Bifi), which are considered atypical for healthy adults in the global collection, showed enrichment in TB patients. Notably, most (90%) healthy donors in our cohort had near-zero abundances of these atypical enterosignatures (ES-Esch and ES-Bifi) (**Supplementary Figure 1**) and a long tail of outliers with increased abundance. We therefore used the 90th percentile point of the empirical distribution as a cutoff for downstream analyses. Specifically, we used this cutoff to focus on a subset of TB patients with increased abundances of ES-Esch and ES-Bifi (**Supplementary Figure 1**). To explore potential associations between gut microbiome composition and host clinical characteristics, we performed correlation analysis between enterosignature abundances (ES-Bact, ES-Bifi, ES-Esch, ES-Prev, ES-Firm) and a panel of clinical parameters. Spearman’s rank correlation coefficients were calculated separately for TB patients before treatment (n = 33) and healthy controls (n = 47). For each correlation analysis, the p-value was computed using the cor.test() function in R version 4.5.3. Correlations with p < 0.05 were considered statistically significant and were marked with an asterisk in the corresponding heatmaps.

### 2.4 Bacterial community diversity and principal component analysis

Shannon alpha diversity was estimated using the microbiome R package (Lahti and Shetty, 2017; Microbiome, 2025). Differences in Shannon alpha diversity between patients and controls were tested using the two-sample Kolmogorov-Smirnov test implemented in the ks.test() function of the stats R package (2022). To visualize gut community differences between donors using major axes of microbial variation, we applied principal component analysis (PCA) on the species abundance matrix. To adapt sparse data on relative abundance for principal component analysis, we imputed zeros in the relative abundance table using the cmultRepl() function in the zCompositions R package version 1.4.0-1 (Palarea-Albaladejo and Martín-Fernández, 2015). To account for compositionality, the relative abundance table was transformed using the centered log-ratio (CLR) before PC analysis. PC analysis was carried out using the prcomp() function in the stats R package (, 2022). Cohen’s d effect sizes were calculated using the Evident tool to assess the magnitude of differences in taxonomic and functional microbiome compositions between TB patients and controls (Rahman et al., 2023). The Evident tool is available at https://github.com/biocore/evident. Confidence intervals were computed using the noncentral t−distribution method to provide more accurate interval estimates.

### 2.5 Inference of taxonomic content of the gut microbiome and between-group comparisons

Before downstream analyses, all bacterial taxa observed in a single donor and with relative abundance less than 0.0001 were discarded. The choice of this abundance threshold ensured that we kept rare bacterial species that can impact human disease, as demonstrated in recent work (Chriswell et al., 2022). MetaPhlAn v4.0 tool was used to infer both known bacterial species and strains, as well as uncharacterized bacterial species (Blanco-Míguez et al., 2023). To facilitate comparison with published data, we analyzed the relative abundances of bacterial taxa in sampled groups (TB patients and controls) at different taxonomic levels by aggregating strains and species into genera, families, orders, classes, and phyla. We used linear discriminant analysis with effect size estimation (LEfSe), implemented in the microbial R package v0.0.22, to identify bacterial taxa that distinguish patients from controls. We also calculated the average proportion of each taxon for patients and controls at different taxonomic levels (e.g., phylum and class).

### 2.6 Inference of the metabolic potential of the gut microbiome and between-group comparisons

Metabolic potential (pathways encoded by bacterial genes) of the fecal microbial community was inferred using HUMAnN 3.6 tool with default settings. HUMAnN 3.6 uses species-specific marker genes to pre-screen for bacterial species present in the analyzed metagenome, then identifies bacterial gene families (UniRef gene families) using their reference genomes, and finally aggregates them into pathways. HUMAnN 3.6 applied in this study was based on the ChocoPhlAn 3 database containing 99,200 reference genomes from 16,800 microbial species, UniProt database (as of January 2019) (UniProt Consortium, 2019), and 87.3 million UniRef90 gene families (Suzek et al., 2015). HUMAnN’s default output, the UniRef gene (family) abundances, were aggregated into MetaCyc (Caspi et al., 2020) pathway abundances using the “humann_regroup_table” script.

To identify MetaCyc pathways that are differentially abundant in patients versus controls, we used linear discriminant analysis with effect size estimation (LEfSe), implemented in the microbial R package v0.0.22. For MetaCyc pathways that showed strong enrichment in patients (those with the largest effect size and highest LDA scores), we also inferred the bacterial species and genera that were major contributors to that pathway. To infer contributing bacteria and genera, we used HUMAnN 3.6 “pathabundance.tsv” output files as follows. For each pathway, whenever possible, HUMAnN 3.6 outputs stratified abundances by contributing taxa, attaching species and genus information to the pathway name. For example, “1CMET2-PWY: folate transformations III (E. coli)|Akkermansia|Akkermansia_muciniphila”. We used this information to compute individual taxon contributions. To compare metabolic pathways associated with atypical enterosignatures with those identified in the full cohort, we selected TB patients with elevated abundances of ES-Esch or ES-Bifi, based on the distribution thresholds derived from healthy controls. LEfSe analysis (LDA ≥ 2, adjusted p ≤ 0.05) was performed separately for each subset, and the resulting sets of differentially abundant pathways were compared using a Venn diagram implemented with the eulerr R package v7.1.0.

## 3 Results

### 3.1 Effect size of TB status on microbiome composition

Earlier studies have described taxonomic alterations in TB patients, but without reporting standardized effect size estimates. Effect size estimates are useful to a) understand the strength of the statistical association between the studied disease and the gut microbiome composition; b) facilitate comparison between studies using standardized measures. We used Cohen’s d to assess the effect size of tuberculosis on the key microbiome readouts (Rahman et al., 2023), namely, principal components (PCs) of taxonomic composition variation and principal components of metabolic pathway variation observed in the studied cohort. As a rule of thumb, Cohen’s d values of 0.2, 0.5, and 0.8 are considered as “small,” “medium,” and “large” effect sizes, respectively (Rahman et al., 2023).

The effect size estimated in our case-control cohort suggests that tuberculosis has a strong association with the taxonomic composition, reflected in marked differences from controls as summarized by PC1 (Cohen’s d = 3.855; 95% CI: 3.12–4.59) but not by PC2 (Cohen’s d = 0.274; 95% CI: -0.17 to 0.72) (**Supplementary Figure 2**, **Supplementary Table 1**). The observed strong difference in species content was captured as the top axis of taxonomic variation (PC1), which clearly separated patients from controls in the PCA plot (**Supplementary Figure 3C**). According to our estimates, the effect size of tuberculosis on microbiome composition can be larger than those reported for chronic inflammatory diseases (Cohen’s d ~ 0.2) (Rahman et al., 2023). However, direct comparison of PCA−based Cohen’s d across studies is complicated because these values depend on preprocessing, feature space, and the variance structure of each dataset. Nevertheless, we note that while effect size estimates are meant to compare studies, they can be affected by unaccounted differences in dataset characteristics.

Next, effect size estimates also suggest that there is a notable difference in pathway abundance between patients and controls, as summarized by PC1 and PC2 (Cohen’s d = 1.336; 95% CI: 0.84–1.83 and Cohen’s d = 1.208; 95% CI: 0.70–1.72, respectively) (**Supplementary Figures 2E, F**). These two top principal components (PCs) of metabolic pathway variation jointly separated TB patients from controls in the PCA analysis (**Supplementary Figure 3С**).

We compared the demographic characteristics of patients with those of healthy donors before their inclusion in the study cohort. **Supplementary Table 2** presents the baseline characteristics of all participants. We found no significant difference in sex between the TB patients and controls (p > 0.05, **Supplementary Table 2**), with male TB patients slightly outnumbering females (57.6% vs. 42.4%). However, a significant difference in age was observed (p < 0.001). The median age of TB patients was 41 years (IQR 36–53), compared to 36 years (IQR 29.5–40) in the control group. For the recruited patients, no information was collected on body mass index, smoking status, lifestyle factors, diet, socioeconomic status, or long-term antibiotic use; therefore, we could not evaluate the effects of these factors on microbiome composition.

### 3.2 Atypical ecological guilds in patients’ fecal microbiome

**Figure 1:**
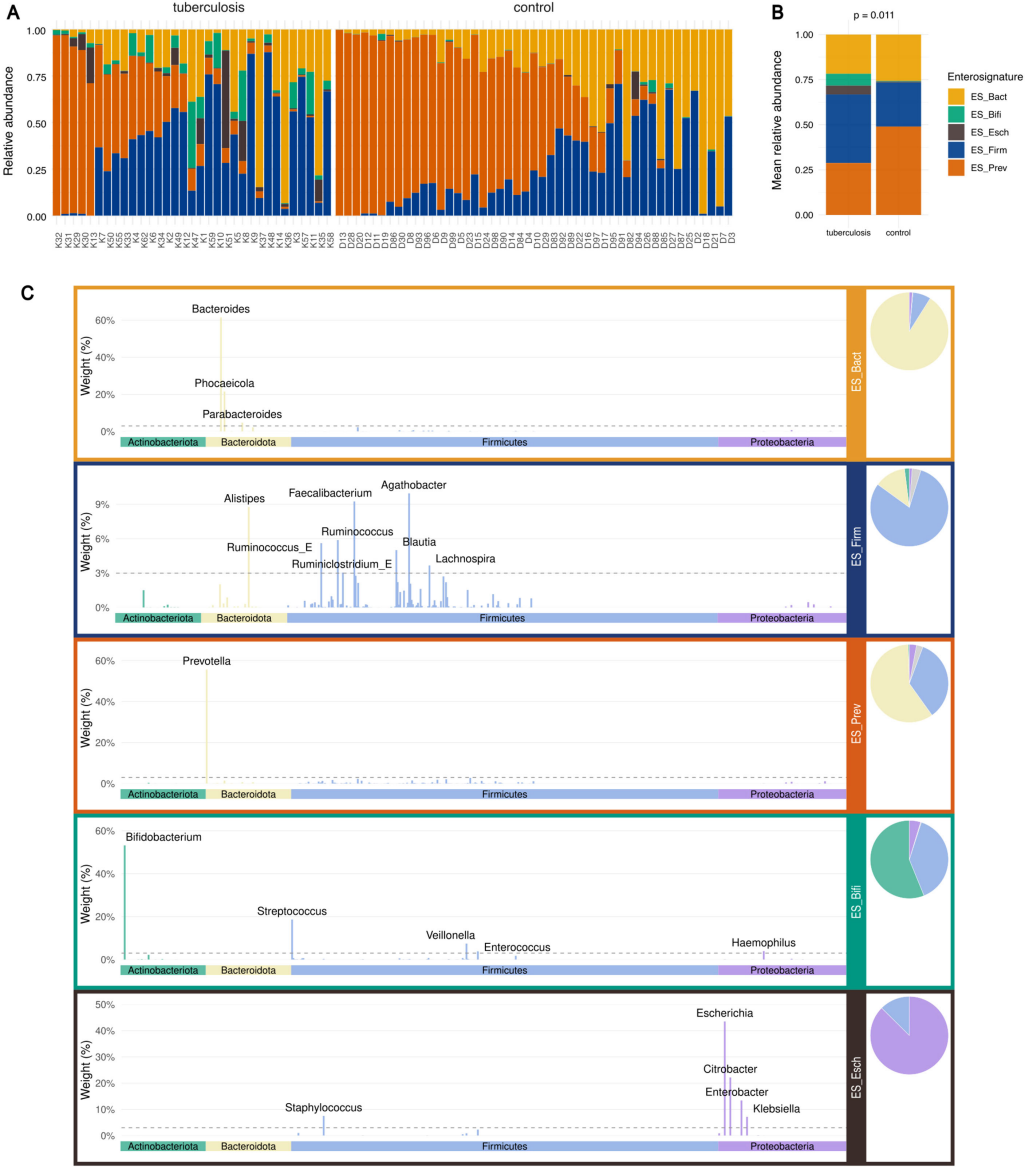
Enterosignature composition of gut microbiomes and member species of enterosignatures. **(A)** The relative proportions of the five ESs in gut microbiomes of TB patients and controls. **(B)** Average proportion of the five ESs in TB patients and controls. Difference in average ES composition between patients and controls was tested using PERMANOVA test (after converting proportions to Aitchison distances) **(С)** Bacterial taxa (at genus-level) defining enterosignatures and their weights pre-computed by NMF method based on the 5,230 fecal metagenomes of the GMR panel.

Bacteria live in communities, and to better understand their community-level functions, we predicted the composition of ecological guilds (enterosignatures) in the analyzed gut microbiomes using a pre-computed five enterosignature model (see Materials and Methods). Gut microbiomes in our donors were composed of five globally observed ESs: ES-Bact (primarily characterized by the genera *Bacteroides* and *Phocaeicola*), ES-Bifi (*Bifidobacterium* and *Streptococcus*), ES-Prev (*Prevotella*), ES-Esch (*Escherichia* and *Citrobacter*), and ES-Firm (genera within the phylum *Firmicutes*). While ESs are labeled by the dominating genera, in reality, they are composed of multiple co-occurring genera in unique combinations (**Figure 1C**). Often, one or two genera dominate the structure of the ecological guild (for instance, ES-Prev (*Prevotella*)), but some ESs consist of multiple genera (**Figure 1C**). Thus, multiple members of the same ecological guild co-occur and are thought to collaborate to exploit the resources of the same ecological niche (Frioux et al., 2023).

Healthy controls in the study harbored various combinations of three primary enterosignatures (ES-Bact, ES-Prev, and ES-Firm) (**Figure 1A**), and these ESs also dominated the fecal microbiomes of the worldwide collection of healthy adults in the GMR panel (Frioux et al., 2023). On average, gut microbes of these three enterosignatures (ES-Bact, ES-Prev, and ES-Firm) constitute 99% of the fecal microbiome of any given healthy individual (**Figure 1B**). Combinations of these enterosignatures are believed to reflect multiple ecological niches within the human gut (Frioux et al., 2023). Notably, two atypical enterosignatures, ES-Bifi and ES-Esch, were almost absent in healthy donors, with few exceptions, such as D19, D94, and D88. In contrast, these two atypical ecological guilds (ES-Bifi and ES-Esch) occurred more frequently in the gut microbiomes of TB patients, who had altered proportions of the primary enterosignatures (PERMANOVA test, p = 0.011) (**Figures 1A, B**). ES-Bifi was detected in 75.8% of samples (25 of 33), whereas ES-Esch was detected in 39% (13 of 33). Indeed, high abundance of ES-Bifi (6.6% vs. 0.4%, p = 1.1 × 10^-11) and ES-Esch (4.9% vs. 0.6%, p = 2.1 × 10^-9) were distinctive features of the patient’s gut community. In addition, primary ESs had altered abundance compared with healthy donors: depleted abundance of ES-Prev (28.8% vs. 49%) and elevated abundance of ES-Firm in some patients, including K8 and K48 (38% vs. 24.4%, p = 0.04). Overall, gut microbiomes in TB patients have two distinctive features - an unusually high abundance (more than 75% of the donor’s fecal microbiome) of ES-Firm in some patients, and relatively high abundance of two atypical enterosignatures, ES-Bifi, and ES-Esch, that are characteristic of gut microbiome in infants, sick individuals, and older individuals (Frioux et al., 2023) (Guner et al., 2009). High proportions of ES-Firm (> 70%) or tendency to dominate the gut community (monodominance) are rare among healthy adults. A tendency toward monodominance (more than 75%) by ES-Firm is considered to reflect a transitional state of the microbial community when gut ecosystems are disturbed, for example, by intensive antibiotic therapy. Moreover, it is hypothesized that ES-Firm is unable to form a stable community on its own and often complements other ESs, particularly ES-Bact (Frioux et al., 2023). Indeed, among the analyzed healthy donors, ES-Firm tended to combine with other enterosignatures (**Figure 1A**). In contrast, the two other primary enterosignatures, ES-Bact and ES-Prev, are thought to be capable of forming a stable monodominant microbial community. For example, healthy donors D13, K28, and K20 exhibit ES-Prev monodominance, while samples D18 and D7 exhibit ES-Bact monodominance (**Figure 1A**).

### 3.3 Conventional taxonomic analysis likely reflects fragments of ecological guilds

**Figure 2:**
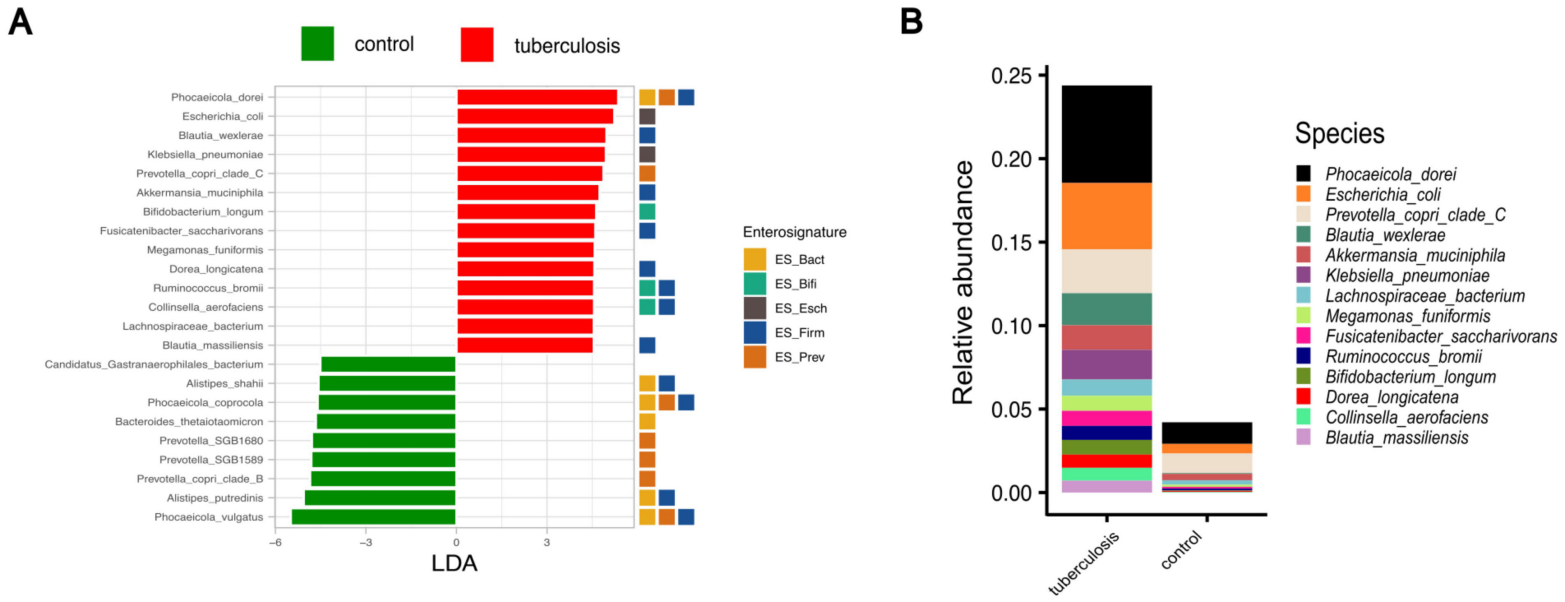
Differentially abundant species between TB patients and healthy controls. **(A)** Barplot with top bacterial species (LDA scores > 4.5) differentially abundant between TB patients and controls. The color-filled squares to the right of the barplot indicate the enterosignature membership. **(B)** Averaged abundances of the top TB-associated species in TB patients and controls (LDA scores > 4.5).

To compare guild-based analysis with the conventional taxonomic approach, we used a popular linear discriminant analysis, LEfSe, as an example. LEfSe analysis identified 274 differentially abundant (adjusted p-value ≤ 0.05 and LDA score ≥ 2.5) taxa (species and subclades defined by MAGs), associated either with TB patients or controls (**Supplementary Table 3**, **Supplementary Figure 4**). While many bacterial species were associated with tuberculosis, each individually showed only a marginal statistical association (LDA scores ranging from 2.0 to 3.0) with the disease. Moreover, most of them are functionally uncharacterized gut commensals, which makes interpretation challenging. To clarify our point further, we highlighted only bacterial species with a strong association (LDA score of 4.5 or higher) to disease (**Figure 2A**). It can be seen that, except for *Escherichia coli* and *Klebsiella pneumoniae*, most TB-enriched species are prevalent human gut commensals, such as *Blautia wexlerae, Prevotella copri clade C*, and *Akkermansia muciniphila* (**Figure 2А**). With results of this kind, one can only conclude that, on average, the TB-enriched gut commensals had higher abundance in patients (**Figure 2B**). To aid interpretation, we reasoned that differentially abundant species represent fragments of underlying ecological guilds in the donor gut community. When we labeled bacterial species by their membership to enterosignatures (colored squares to the right of the barplot in **Figure 2A**), it became apparent that health and disease-associated species were preferentially affiliated to primary adult and atypical enterosignatures, respectively. Thus, most of the health-associated species tended to be members of the three primary adult enterosignatures (ES-Bact, ES-Prev, and ES-Firm) (**Figure 2A**, colored squares to the right of the barplot) while the patient-associated species often belonged to the two atypical microbial guilds (ES-Bifi and ES-Esch) or ES-Firm. One can see that mapping complex taxonomic data to enterosignatures helps identify lower-dimensional ecological units that better correlate with disease or health. Indeed, multiple seemingly unrelated gut commensals associated with patients (positive LDA-scores) represent fragments of two atypical ecological guilds and ES-Firm (**Figure 2A**). We next reasoned that the prevalence of atypical guilds in TB patients is reflected in the increased number of member species, their genes, and encoded metabolic pathways. We therefore inferred microbial genes and pathways encoded by enterosignature members, binned them into respective ecological guilds, and ran differential abundance tests.

### 3.4 Microbial pathways enriched in ES-Esch and ES-Bifi

**Figure 3:**
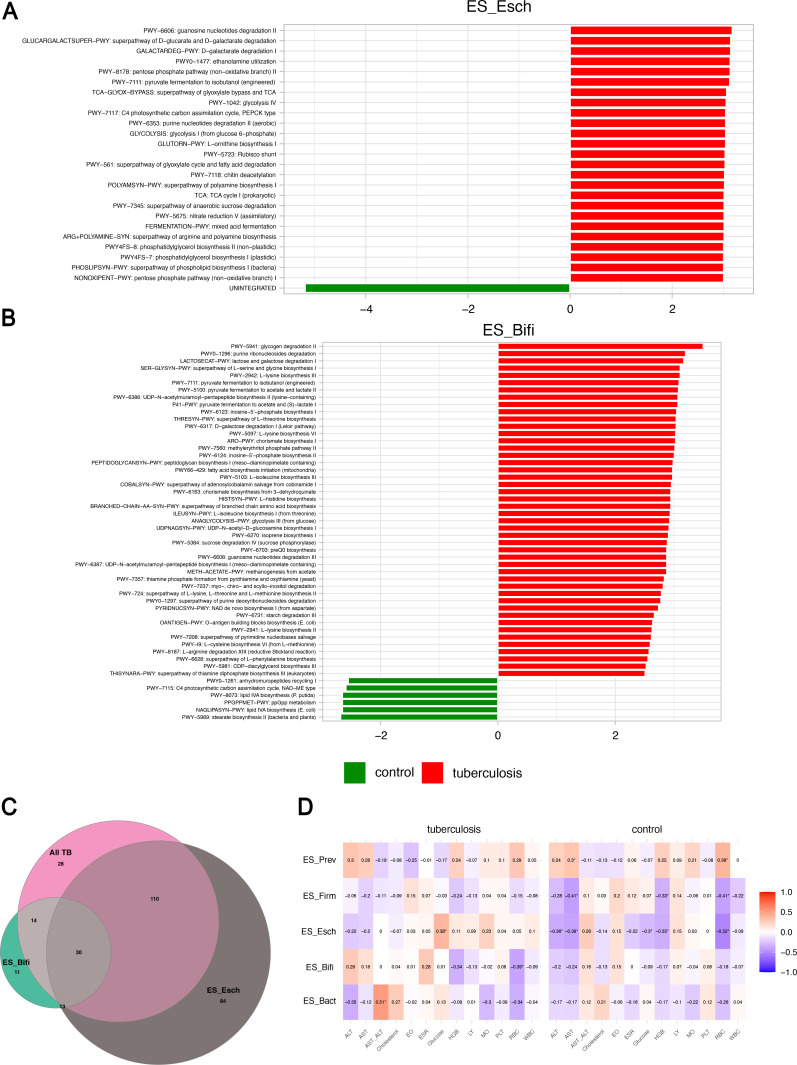
Differential metabolic potential of the ES-Esch and ES-Bifi enterosignatures and their correlations to host (clinical) biomarkers. **(A, B)** Linear discriminant analysis (LDA) effect size scores for metabolic pathways enriched in ES-Esch (LDA index > 3) **(A)** and ES-Bifi (LDA index > 2.5) **(B)** in tuberculosis patients (red) versus their respective healthy controls (green). **(C)** Overlap of metabolic pathways significantly enriched in the overall TB cohort, ES-Esch, and ES-Bifi (LDA ≥ 2, p.adj ≤ 0.05). **(D)** Heatmap showing Spearman сorrelation coefficients between enterosignature relative abundances and blood clinical biomarkers. Red indicates a positive sign of correlation, while blue indicates a negative correlation (P < 0.05). Significant correlations (p < 0.05) are indicated with asterisks. ALT, alanine transaminase; AST, aspartate transaminase; AST_ALT, ratio between the concentrations of two enzymes, aspartate transaminase (AST) and alanine transaminase (ALT); EO, eosinophils; ESR, an erythrocyte sedimentation rate; HGB, hemoglobin; LY, lymphocytes; MO, monocytes; PLT, platelets; RBC, red blood cells; WBC, white blood cells.

Most TB patients demonstrate higher occurrence and abundance of atypical ecological guilds (ES-Esch and ES-Bifi) in addition to three primary ESs. We attempted to highlight metabolic pathways that are overrepresented in TB patients’ microbiomes due to the higher abundance of ES-Esch and ES-Bifi member species. We selected patients with elevated abundances of ES-Esch and ES-Bifi, ensuring that observed abundances were rarely encountered among healthy donors. Most healthy donors (90%) had near-zero abundances of these enterosignatures, and 10% represented a long tail of outliers with increasing abundances (**Supplementary Figure 1**). Assuming that near-zero values are more characteristic of healthy donors, we defined the 90th percentile points of healthy donor distributions as cutoffs: 0.75% for ES-Esch and 0.78% for ES-Bifi. These cutoffs were close to independent estimates for healthy donors in the Human Microbiome Project (HMP): 0.6% for ES-Esch and 1.6% for ES-Bifi (Frioux et al., 2023). Based on these cutoffs, we prepared two subsets of TB patients that had elevated levels of ES-Esch (N = 19) and ES-Bifi (N = 26). These subsets enriched for ES-Esch or ES-Bifi were compared to healthy controls using linear discriminant analysis. Since the compared cases and controls also had primary enterosignatures in their gut microbiomes (See **Figure 1A**), we retained only pathways encoded by the member species of atypical enterosignatures. For this, we filtered the HUMAnN-predicted (Metacyc) pathways encoded by the member species of ES-Esch and ES-Bifi (Member species belong to the genera shown in **Figure 1C**). In this manner, we aimed at delineating metabolic pathways that are 1) encoded by ES−Esch− and ES−Bifi−associated gut microbes and 2) enriched in patients but have trace abundance in controls (controls have near-zero abundance of atypical guilds). Linear discriminant analysis (LDA) identified a large number of differentially abundant metabolic pathways (LDA score ≥ 2.5), essentially driven by higher abundances of ES-Esch and ES-Bifi in patients and trace levels in controls (**Figures 3A, B**; **Supplementary Figures 5**, **6**). Here, differentially abundant pathways reflect the abundance (number) of bacterial gene carriers rather than enhanced pathway activity. For downstream analyses, we focused on pathways involved in energy acquisition, since gut microbial composition is primarily driven by nutrient-acquisition strategies (Muramatsu and Winter 2024). As expected, member species of both enterosignatures feature two versions of anaerobic glycolysis, which are central for energy acquisition (Embden-Meyerhof-Parnas pathway: ANAGLYCOLYSIS-PWY) and (glycolysis II from fructose 6-phosphate: PWY-5484). Next, as would be expected for gut microbes, genes are present for different variants of fermentation (such as mixed acid fermentation: FERMENTATION-PWY; homolactic fermentation: ANAEROFRUCAT-PWY; pyruvate fermentation to acetate and (S)-lactate I: P41-PWY; hexitol fermentation to lactate, formate, ethanol and acetate: P461-PWY, and pyruvate fermentation to acetate and lactate II: PWY-5100) (See full list in **Supplementary Figures 5**, **6** highlighted in yellow, **Supplementary Table 4**, tabs: ES-Esch and ES-Bifi). Other pathways serve biosynthetic processes that promote active bacterial growth and division (**Supplementary Figure 7**). For instance, amino acid biosynthesis (ARGSYN-PWY, GLUTORN-PWY, ARGSYNBSUB-PWY, PWY-5154), essential for protein synthesis in rapidly dividing cells and pathways for the biosynthesis of essential vitamins and cofactors (PWY-5918, PWY-7357, COBALSYN-PWY), and finally pathways for nucleotide and cell envelope components (PEPTIDOGLYCANSYN-PWY, PWY0-1586, PWY-6385).

### 3.5 Differences in pathway abundance between ES-Esch and ES-Bifi

The member species of ES-Esch and ES-Bifi exhibit notable differences. For instance, ES-Bifi featured pathways for lactose utilization (lactose degradation I: LACTOSECAT-PWY) and its subsequent fermentation to lactate and acetate (pyruvate fermentation to acetate and (S)-lactate I: P41-PWY; pyruvate fermentation to acetate and lactate II: PWY-5100). In addition, one can notice that an increase in ES-Bifi brings capacity for (PWY-5941) for endogenous glycogen degradation (**Figure 3B**; **Supplementary Table 4**). This pathway enables *Bifidobacteria* to utilize simple sugars and endogenous glycogen stores (PWY-5941, PWY-5484) when typical dietary substrates (plant-derived polysaccharides, milk oligosaccharides) are absent. In contrast, ES-Esch looks more versatile in terms of glycolytic and mixed fermentation pathways (mixed acid fermentation: FERMENTATION-PWY; hexitol fermentation to lactate, formate, ethanol, and acetate: P461-PWY), indicating the capacity to efficiently utilize sugars and sugar derivatives, including D-galactarate (D-galactarate degradation I: GALACTARDEG-PWY), D-glucarate (superpathway of D-glucarate and D-galactarate degradation: GLUCARGALACTSUPER-PWY), and hexitols (superpathway of hexitol degradation (bacteria): HEXITOLDEGSUPER-PWY). Moreover, ES−Esch member-species featured metabolic pathways for assimilatory nitrate reduction (PWY-5675) and sulfate assimilation (SULFATE-CYS-PWY) (**Supplementary Table 4**).

### 3.6 Metabolic pathways enriched in TB patient microbiomes are driven by ES-Esch and ES-Bifi

Differential abundance tests typically compare patients and controls without binning or attributing compared pathways to species of origin or their functional guilds. Here, we first performed a typical differential analysis comparing the full TB cohort to healthy controls, and then explored whether the identified TB-associated pathways could be attributed to ES-Bifi- and ES-Esch. In the full case-control cohort, we identified 185 metabolic pathways that were differentially abundant in TB patients or healthy controls (adjusted p-value ≤ 0.05, LDA score ≥ 2). Of these, 182 pathways were associated with TB patients and only three with healthy controls (**Supplementary Table 4**, **Supplementary Figure 8**). Top pathways associated with TB patient microbiome included pyruvate fermentation to isobutanol (PWY-7111), glycogen degradation (PWY-5941) and biosynthesis (GLYCOGENSYNTH-PWY), the pentose phosphate pathway (non-oxidative branch I and II: NONOXIPENT-PWY, PWY-8178), sucrose biosynthesis II (PWY-7238), pyruvate fermentation to acetate and lactate II (PWY-5100), and glycolysis (GLYCOLYSIS, ANAGLYCOLYSIS-PWY, PWY-5484), as well as numerous pathways involved in amino acid and nucleotide biosynthesis (GLUTORN-PWY, ARGSYNBSUB-PWY, ARGSYN-PWY, PWY-6608, PWY0-1296) (**Supplementary Table 4**). We cross-checked the full list of TB-associated pathways against those previously attributed to ES-Esch and ES-Bifi (**Figures 3A, B**, **Supplementary Figures 5**, **6**). Cross-checking showed that most of the differentially abundant pathways in the full TB cohort can be explained by pathways attributable to the two atypical ESs (see black and turquoise squares in **Supplementary Figure 8**). Thus, microbial pathways attributable to member species of ES-Bifi- and ES-Esch can explain what can be different in terms of microbial function in TB patients’ gut microbiomes. This is not surprising, given that differential abundance analysis essentially “cancels out” everything shared between TB and healthy controls. In this case, shared features represent the full spectrum of microbial functions encoded by members of the three primary enterosignatures (ES-Bact, ES-Prev, and ES-Firm) shared by patients and controls (**Figure 1A**).

To further evaluate commonalities and differences between the two inference approaches (the bulk case-control versus binning to enterosignatures), we constructed a Venn diagram (**Figure 3C**). Notably, 154 out of 180 (85%) differentially abundant pathways identified by the conventional case-control analysis can be explained by metabolic pathways associated with the two atypical enterosignatures. Next, metabolic pathways attributed to ES-Esch (in total, 237 pathways in **Supplementary Table 5**) explain the largest fraction (140 out of 180) of the pathways identified as TB-associated using the conventional case-control analysis. In the meantime, pathways attributed to ES-Bifi explain only 44 out of 180 of the 180 total TB-associated pathways in the conventional analysis. Taken together, our results suggest that seemingly modest shifts in the abundance of atypical enterosignatures (**Figure 1A**) shape most of the genetically encoded microbial function that is different in the patient’s gut microbiome.

Assuming that ecological guilds better represent microbial functions, we next searched for associations between the identified enterosignatures and clinical blood parameters. We performed a Spearman correlation analysis (**Figure 3D**), which revealed notable associations with clinical relevance. For instance, ES-Esch showed a positive correlation with glucose (ρ = 0.38, p < 0.05), while ES-Bifi showed a negative correlation with red blood cell count (ρ = -0.39, p < 0.05). Interestingly, ES-Bact, a primary enterosignature in the healthy cohort, demonstrated the strongest positive correlation with the AST/ALT ratio (ρ = 0.51, p < 0.05), a biomarker of liver function. Taken together, our analyses suggest that enterosignatures represent a biologically meaningful unit to reduce the complexity of the human gut microbiome and a tool to recognize sharper patterns behind noisy taxonomic and functional diversity.

## 4 Discussion

In this study, we applied the concept of enterosignatures — ecological guilds of co-occurring microbial species — to analyze alterations in the gut microbiota of TB patients. Fecal samples from most participants predominantly contained three enterosignatures: ES-Bact, ES-Prev, and ES-Firm. Based on a global sample of gut microbiomes, Frioux et al. (2023) showed that ES-Bact and ES-Prev constitute the stable core of a healthy human microbiome. In contrast, ES-Firm is likely successional and was rarely found in isolation within an individual microbiome (Frioux et al., 2023). A distinctive feature of fecal microbiome from TB patients was a statistically significant enrichment (higher occurrence and abundance) of two atypical enterosignatures — ES-Bifi and ES-Esch — which were nearly absent in healthy donors. This observed increase in two ecological guilds suggests that the intestines of TB patients differ as an ecological niche from those of healthy individuals. We hypothesize that the increase in atypical guilds reflects the microbiota’s response to systemic inflammation during active TB. Host inflammation is known to elevate concentrations of oxygen and other host-derived electron acceptors in the intestinal lumen by disrupting mucosal hypoxia, thereby creating a selective advantage for facultative anaerobes (Lee et al., 2024; Litvak et al., 2018). Transition from anoxic to a microaerobic environment, therefore, favors facultative anaerobes capable of utilizing flux of electron acceptors resulting from inflammation. In this context, our finding of enrichment of ES-Esch — a guild representing facultative anaerobes (primarily *E. coli*) — in patients with TB could be interpreted as a sign of this disrupted anoxic environment in the colon, a phenomenon that is currently relatively well understood mechanistically (Lee et al., 2024). Notably, the nitrate (PWY-5675) and sulfate (SULFATE-CYS-PWY) assimilation pathways encoded by ES-Esch member species can further benefit this guild in the inflamed intestines of TB patients.

Another interesting observation is that the ecological guild of ES-Esch species correlates with elevated blood glucose. While this statistical correlation does not necessarily imply a causal link, we note that the bacterial genera comprising this ecological guild include prominent facultative anaerobic saccharolytic species. For example, *E. coli* and *Staphylococcus aureus* (André et al., 2021). Indeed, the metabolic profile inferred for ES-Esch in the gut microbiome of TB patients highlights a large number of metabolic pathways for degrading various simple sugars. Although direct measurements of luminal glucose or other simple sugars are lacking, we predicted the abundance of glycolytic and fermentative pathways due to ES-Esch member species, which indirectly indicate the availability of simple sugars in the patients’ intestines. Host inflammation, which accompanies active TB, is known to alter intestinal permeability and cause malabsorption of sugars (Peuhkuri et al., 2010; Koepsell 2020). The resulting increase in the availability of simple sugars in the colonic lumen can potentially boost the expansion of saccharolytic facultative anaerobes that constitute the ES-Esch ecological guild. Further studies with metabolomic readouts are needed to explore the proposed link between ES-Esch in the intestines of TB patients and possible alterations in the flux of simple sugars and inflammation-associated electron acceptors.

An intriguing finding of our study is the elevated abundance of ES-Bifi in the gut microbiome of TB patients. This result may seem counterintuitive if we equate the entire ecological guild to its single member genus, *Bifidobacterium*, which is not true. Indeed, *Bifidobacterium* members are widely known for their health-promoting properties and are used as a probiotic (J. Chen et al., 2021) (Hidalgo-Cantabrana et al., 2017) (Ku et al., 2024) (O’Callaghan and van Sinderen, 2016). The association of the *Bifidobacterium* genus with TB patients has been controversial. Some studies have reported decreased abundance (Y. Wang et al., 2022), while others have demonstrated an increase in *Bifidobacteria spp—*abundance in the gut microbiome (Han et al., 2024). However, a recent large-scale study involving 1803 Japanese individuals supports the association of *Bifidobacterium* with inflammatory conditions. Specifically, this study reported that a *Bifidobacterium*-enriched gut microbiome was significantly associated with a higher prevalence of inflammatory bowel disease, cardiovascular disease, and diabetes (Takagi et al., 2022).

Furthermore, another study identified specific *Bifidobacteriaceae* taxa as potential biomarkers of disease/health status (Pasolli et al., 2024). Consistent with association with inflammation, patients with drug-resistant TB demonstrated higher levels of *Enterobacteriales, Bifidobacteriales, Verrucomicrobiales*, and *Lactobacillales* within the coliform flora (Shi et al., 2022). Conflicting findings regarding the role of *Bifidobacterium* in health and disease can be resolved if we understand microbial functions at the guild level that result from multi-species interaction. Apart from *Bifidobacterium*, ES-Bifi contains notable pathogens and opportunistic species, including members of *Streptococcus, Veillonella, Haemophilus*, and *Enterococcus*. The overall community function may explain why this ecological guild is associated with adverse health conditions. The net effect of Bifidobacterium likely depends on the community and its abundance, which we find must be near zero among healthy adult donors.

Similar to ES-Esch, ES-Bifi represents a guild of facultative anaerobes. Unlike primary ESs such as ES-Bact and ES-Prev — which are dominated by obligate anaerobes under strictly anoxic conditions — ES-Bifi retains metabolic flexibility and the ability to grow in the presence of oxygen. Analysis of the ES-Bifi metabolic profile revealed enrichment of pathways associated with energy production from various sources, along with increased homolactic fermentation leading to lactate formation (P41-PWY, PWY-5100). Lactate, produced through bacterial fermentation in the gastrointestinal tract, plays distinct metabolic and signaling roles in gut dysbiosis and certain metabolic disorders (Li et al., 2025a). Notably, excess D-lactate produced by certain intestinal bacteria can enter the bloodstream and cause metabolic acidosis, particularly in patients with malabsorption syndrome (Levitt and Levitt, 2020; Li et al., 2025). In the context of tuberculosis, elevated serum lactate levels have long been associated with higher mortality during TB treatment (Im et al., 2021; Ntambwe and Maryet, 2012). Mechanistically, lactate, on the one hand, improves *M. tuberculosis* clearance in already infected macrophages by promoting autophagy.

On the other hand, it significantly suppresses both TNF-α and IFN-γ but leaves IL6 and IL10 unaffected, thereby interfering with the enhancement of anti-MTB activity (Ó Maoldomhnaigh et al., 2021). Moreover, lactate specifically upregulates matrix metalloproteinases that contribute to lung destruction in tuberculosis (Whittington et al., 2023), a process known to increase morbidity and mortality during TB treatment (Ong et al., 2014). Another interesting finding is the high abundance of the glycogen degradation pathway (PWY-5941) in ES-Bifi. This pathway, which exhibited the highest LDA score among all significantly enriched pathways in this guild, could indicate that bacteria within ES-Bifi are actively mobilizing their endogenous glycogen reserves.

In this study, we also attempted to explore how guild-based binning compares with conventional analyses, in which bacterial taxa and metabolic pathways are analyzed as individual units of the gut microbiome. For example, using a popular LEfSe analysis, we identified a broad array of differentially abundant bacterial taxa in TB patients, but the biological significance of these findings remained elusive, as many of the patient-enriched bacteria are common gut commensals that lack functionally established pathogenic roles. However, when we mapped bacterial species to enterosignatures, it became apparent that health-associated taxa belonged to the primary adult ESs (ES-Bact, ES-Prev, and ES-Firm). In contrast, patient-associated taxa belonged to the atypical ESs ES-Bifi and ES-Esch. A similar tendency was observed when analyzing differentially abundant metabolic pathways. In the gut microbiome of TB patients, the top-ranked pathways were predominantly associated with ES-Esch and ES-Bifi. These findings demonstrate that the metabolic potential of the gut microbiome in TB patients is primarily shaped by the unusually high levels of the two atypical enterosignatures. We conclude that conventional taxonomic and functional profiling of the gut microbiome, while informative for distinguishing TB patients from healthy controls, provides limited insight into the sharper structuring evident within ecological guilds. Moreover, without guild inference, it would be challenging to appreciate guild-level functions, such as energy-acquisition strategies that reflect microbial responses to the intestinal milieu, which provide an indirect insight into intestinal alterations in patients. Thus, the enterosignature-based approach represents a biologically meaningful and useful way to summarize the extremely complex functions of the microbiome.

## 5 Limitations of the study

Our study has several limitations. First, the small sample size and single geographic source of the study population limit the generalizability of our findings. The gut microbiota is influenced by regional environmental conditions, lifestyle, and additional factors such as smoking, BMI, diet, metabolic comorbidities, and antibiotic exposure before diagnosis; therefore, large, multi-center population cohort studies are needed to validate current findings further. Second, we observed a slight but statistically significant difference in age between TB patients and controls. Given that age is a known confounder of gut microbiota composition, we cannot exclude the possibility that observed differences in enterosignature distribution may be partially affected by age rather than TB status. Next, we note that our inferences about microbial metabolic functions are based on computationally predicted genes and pathways from metagenomic sequencing data rather than direct metabolomic readouts. Therefore, metabolic pathways must be understood as genetically encoded potential, and our working hypotheses need experimental confirmation using metabolomic approaches. In conclusion, enrichment of atypical enterosignatures in TB status represents a statistical signal, and whether this pattern reflects microbial response to disease or cause requires further investigation.

## Data Availability

The datasets presented in this study can be found in online repositories. The names of the repository/repositories and accession number(s) can be found below: https://www.ncbi.nlm.nih.gov/, PRJNA1061168 https://www.ncbi.nlm.nih.gov/, PRJNA1366497.

## References

[B1] André A. C. Debande L. Marteyn B. S. (2021). The selective advantage of facultative anaerobes relies on their unique ability to cope with changing oxygen levels during infection. Cell. Microbiol. 23 (8), e13338. doi: 10.1111/cmi.13338 33813807

[B2] Arumugam M. Raes J. Pelletier E. Le Paslier D. Yamada T. Mende D. R. . (2011). Enterotypes of the human gut microbiome. Nature 473, 174–180. doi: 10.1038/nature09944 21508958 PMC3728647

[B3] Benson G. (1999). Tandem repeats finder: a program to analyze DNA sequences. Nucleic Acids Res. 27, 573–580. doi: 10.1093/nar/27.2.573 9862982 PMC148217

[B4] Blanco-Míguez A. Beghini F. Cumbo F. McIver L. J. Thompson K. N. Zolfo M. . (2023). Extending and improving metagenomic taxonomic profiling with uncharacterized species using MetaPhlAn 4. Nat. Biotechnol. 41 (11), 1633–1644. doi: 10.1038/s41587-023-01688-w 36823356 PMC10635831

[B5] Bolger A. M. Lohse M. Usadel B. (2014). Trimmomatic: a flexible trimmer for Illumina sequence data. Bioinf. (Oxford. England.) 30, 2114–2120. doi: 10.1093/bioinformatics/btu170 24695404 PMC4103590

[B6] Budden K. F. Gellatly S. L. Wood D. L. A. Cooper M. A. Morrison M. Hugenholtz P. . (2017). Emerging pathogenic links between microbiota and the gut–lung axis. Nat. Rev. Microbiol. 15, 55–63. doi: 10.1038/nrmicro.2016.142 27694885

[B7] Burke C. Steinberg P. Rusch D. Kjelleberg S. Thomas T. (2011). Bacterial community assembly based on functional genes rather than species. PNAS 108, 14288–14293. doi: 10.1073/pnas.1101591108 21825123 PMC3161577

[B8] Campbell C. Kandalgaonkar M. R. Golonka R. M. Yeoh B. S. Vijay-Kumar M. Saha P. (2023). Crosstalk between gut Microbiota and host immunity: Impact on inflammation and immunotherapy. Biomedicines 11, 294. doi: 10.3390/biomedicines11020294 36830830 PMC9953403

[B9] Caspi R. Billington R. Keseler I. M. Kothari A. Krummenacker M. Midford P. E. . (2020). The MetaCyc database of metabolic pathways and enzymes - a 2019 update. Nucleic Acids Res. 48, D445–D453. doi: 10.1093/nar/gkz862 31586394 PMC6943030

[B10] Chen J. Chen X. Ho C. L. (2021). Recent development of probiotic Bifidobacteria for treating human diseases. Front. Bioeng. Biotechnol. 9, 770248. doi: 10.3389/fbioe.2021.770248 35004640 PMC8727868

[B11] Chen S. Zhou Y. Chen Y. Gu J. (2018). fastp: an ultra-fast all-in-one FASTQ preprocessor. Bioinformatics 34, i884–i890. doi: 10.1093/bioinformatics/bty560 30423086 PMC6129281

[B12] Chriswell M. E. Lefferts A. R. Clay M. R. Hsu A. R. Seifert J. Feser M. L. . (2022). Clonal IgA and IgG autoantibodies from individuals at risk for rheumatoid arthritis identify an arthritogenic strain of Subdoligranulum. Sci. Transl. Med. 14, eabn5166. doi: 10.1126/scitranslmed.abn5166 36288282 PMC9804515

[B13] de Gaay Fortman D. P. E. Kullberg R. F. J. Wiersinga W. J. Haak B. W. (2026). Dynamics of the gut and lung microbiota in severe infections: from observational studies to therapeutic strategies. Clin. Microbiol. Infection.: Off. Publ. Eur. Soc. Clin. Microbiol. Infect. Dis. 32 (7), 1067–1074. doi: 10.1016/j.cmi.2026.02.016 41748016

[B14] Frioux C. Ansorge R. Özkurt E. Ghassemi Nedjad C. Fritscher J. Quince C. . (2023). Enterosignatures define common bacterial guilds in the human gut microbiome. Cell. Host Microbe 31, 1111–1125.e6. doi: 10.1016/j.chom.2023.05.024 37339626

[B15] Guner Y. S. Malhotra A. Ford H. R. Stein J. E. Kelly L. K. (2009). Association of Escherichia coli O157:H7 with necrotizing enterocolitis in a full-term infant. Pediatr. Surg. Int. 25, 459–463. doi: 10.1007/s00383-009-2365-3 19396605

[B16] Han M. Wang X. Zhang J. Su L. Ishaq H. M. Li D. . (2024). Gut bacterial and fungal dysbiosis in tuberculosis patients. BMC Microbiol. 24 (1), 141. doi: 10.1186/s12866-024-03275-8 38658829 PMC11044546

[B17] Hidalgo-Cantabrana C. Delgado S. Ruiz L. Ruas-Madiedo P. Sánchez B. Margolles A. (2017). Bifidobacteria and their health-promoting effects. Microbiol. Spectr. 5, 10.1128/microbiolspec.bad-0010-2016. doi: 10.1128/microbiolspec.bad-0010-2016 28643627 PMC11687494

[B18] Hildebrand F. Gossmann T. I. Frioux C. Özkurt E. Myers P. N. Ferretti P. . (2021). Dispersal strategies shape persistence and evolution of human gut bacteria. Cell Host Microbe 29 (7), 1167–1176.e9. doi: 10.1016/j.chom.2021.05.008 34111423 PMC8288446

[B19] Hu Y. Feng Y. Wu J. Liu F. Zhang Z. Hao Y. . (2019). The gut microbiome signatures discriminate healthy from pulmonary tuberculosis patients. Front. Cell. Infect. Microbiol. 9, 90. doi: 10.3389/fcimb.2019.00090 31001490 PMC6456665

[B20] Im J. H. Lee J.-S. Chung M.-H. Kwon H. Y. Lee M.-J. Baek J. H. (2021). Effect of a serum lactate monitoring recommendation policy on patients treated with linezolid. Medicine 100, e23790. doi: 10.1097/MD.0000000000023790 33429740 PMC7793345

[B21] Jiang X. Langille M. G. I. Neches R. Y. Elliot M. Levin S. A. Eisen J. A. . (2012). Functional biogeography of ocean microbes revealed through non-negative matrix factorization. PloS One 7, e43866. doi: 10.1371/journal.pone.0043866 23049741 PMC3445553

[B22] Khan N. Vidyarthi A. Nadeem S. Negi S. Nair G. Agrewala J. N. (2016). Alteration in the gut Microbiota provokes susceptibility to tuberculosis. Front. Immunol. 7, 529. doi: 10.3389/fimmu.2016.00529 27965663 PMC5124573

[B23] Koepsell H. (2020). Glucose transporters in the small intestine in health and disease. Pflugers Arch. 472 (9), 1207–1248. doi: 10.1007/s00424-020-02439-5 32829466 PMC7462918

[B24] Ku S. Haque M. A. Jang M. J. Ahn J. Choe D. Jeon J. I. . (2024). The role of Bifidobacterium in longevity and the future of probiotics. Food Sci. Biotechnol. 33, 2097–2110. doi: 10.1007/s10068-024-01631-y 39130652 PMC11315853

[B25] Lahti L. Shetty S. (2017). Tools for microbiome analysis in R. Available online at: http://microbiome.github.com/microbiome (Accessed July 7, 2025).

[B26] Lee J.-Y. Bays D. J. Savage H. P. Bäumler A. J. (2024). The human gut microbiome in health and disease: time for a new chapter? Infect. Immun. 92, e0030224. doi: 10.1128/iai.00302-24 39347570 PMC11556149

[B27] Levitt M. D. Levitt D. G. (2020). Quantitative evaluation of D-lactate pathophysiology: New insights into the mechanisms involved and the many areas in need of further investigation. Clin. Exp. Gastroenterol. 13, 321–337. doi: 10.2147/CEG.S260600 32982363 PMC7490090

[B28] Li W. Huang Y. Tong S. Wan C. Wang Z. (2024). The characteristics of the gut microbiota in patients with pulmonary tuberculosis: A systematic review. Diagn. Microbiol. Infect. Dis. 109, 116291. doi: 10.1016/j.diagmicrobio.2024.116291 38581928

[B29] Li X. V. Leonardi I. Iliev I. D. (2019b). Gut mycobiota in immunity and inflammatory disease. Immunity 50, 1365–1379. doi: 10.1016/j.immuni.2019.05.023 31216461 PMC6585451

[B30] Li J. Ma P. Liu Z. Xie J. (2025). L- and D-Lactate: unveiling their hidden functions in disease and health. Cell. Comm. Signal.: CCS. 23, 134. doi: 10.1186/s12964-025-02132-z 40075490 PMC11905701

[B31] Li W. Zhu Y. Liao Q. Wang Z. Wan C. (2019a). Characterization of gut microbiota in children with pulmonary tuberculosis. BMC Pediatr. 19, 445. doi: 10.1186/s12887-019-1782-2 31735171 PMC6859623

[B32] Libertucci J. Young V. B. (2019). The role of the microbiota in infectious diseases. Nat. Microbiol. 4, 35–45. doi: 10.1038/s41564-018-0278-4 30546094

[B33] Litvak Y. Byndloss M. X. Bäumler A. J. (2018). Colonocyte metabolism shapes the gut microbiota. Sci. (New. York. N.Y.) 362, eaat9076. doi: 10.1126/science.aat9076 30498100 PMC6296223

[B34] Louca S. Jacques S. M. S. Pires A. P. F. Leal J. S. Srivastava D. S. Parfrey L. W. . (2016). High taxonomic variability despite stable functional structure across microbial communities. Nat. Ecol. Evol. 1, 15. doi: 10.1038/s41559-016-0015 28812567

[B35] Luo M. Liu Y. Wu P. Luo D.-X. Sun Q. Zheng H. . (2017). Alteration of gut microbiota in patients with pulmonary tuberculosis. Front. Physiol. 8, 822. doi: 10.3389/fphys.2017.00822 29204120 PMC5698276

[B36] McIver L. J. Abu-Ali G. Franzosa E. A. Schwager R. Morgan X. C. Waldron L. . (2018). bioBakery: a meta’omic analysis environment. Bioinf. (Oxford. England.) 34, 1235–1237. doi: 10.1093/bioinformatics/btx754 29194469 PMC6030947

[B37] Microbiome (2025). Available online at: http://microbiome.github.com/microbiome (Accessed July 7, 2025).

[B38] Muramatsu M. K. Winter S. E. (2024). Nutrient acquisition strategies by gut microbes. Cell Host Microbe 32 (6), 863–874. doi: 10.1016/j.chom.2024.05.011 38870902 PMC11178278

[B39] Ntambwe M. Maryet M. (2012). Tuberculosis and lactic acidosis as causes of death in adult patients from a regional hospital in Johannesburg. Afr. J. Primary. Health Care Family Med. 4 (1), 266. doi: 10.4102/phcfm.v4i1.266

[B40] O’Callaghan A. van Sinderen D. (2016). Bifidobacteria and their role as members of the human gut Microbiota. Front. Microbiol. 7, 925. doi: 10.3389/fmicb.2016.00925 27379055 PMC4908950

[B41] Ó Maoldomhnaigh C. Cox D. J. Phelan J. J. Mitermite M. Murphy D. M. Leisching G. . (2021). Lactate alters metabolism in human macrophages and improves their ability to kill Mycobacterium tuberculosis. Front. Immunol. 12, 663695. doi: 10.3389/fimmu.2021.663695 34691015 PMC8526932

[B42] Ong C. W. M. Elkington P. T. Friedland J. S. (2014). Tuberculosis, pulmonary cavitation, and matrix metalloproteinases. Am. J. Respir. Crit. Care Med. 190, 9–18. doi: 10.1164/rccm.201311-2106PP 24713029 PMC4226026

[B43] Palarea-Albaladejo J. Martín-Fernández J. A. (2015). zCompositions — R package for multivariate imputation of left-censored data under a compositional approach. Chemomet. Intel. Lab. Sys.: An. Int. J. Sponsored. by. Chemomet. Soc. 143, 85–96. doi: 10.1016/j.chemolab.2015.02.019 38826717

[B44] Pasolli E. Mauriello I. E. Avagliano M. Cavaliere S. De Filippis F. Ercolini D. (2024). Bifidobacteriaceae diversity in the human microbiome from a large-scale genome-wide analysis. Cell Rep. 43, 115027. doi: 10.1016/j.celrep.2024.115027 39602306

[B45] Peuhkuri K. Vapaatalo H. Korpela R. (2010). Even low-grade inflammation impacts on small intestinal function. World J. Gastroenterol. 16 9, 1057–1062. doi: 10.3748/wjg.v16.i9.1057 20205274 PMC2835780

[B46] Raguideau S. Plancade S. Pons N. Leclerc M. Laroche B. (2016). Inferring aggregated functional traits from metagenomic data using constrained Non-negative Matrix Factorization: Application to fiber degradation in the human gut Microbiota. PloS Comput. Biol. 12, e1005252. doi: 10.1371/journal.pcbi.1005252 27984592 PMC5161307

[B47] Rahman G. McDonald D. Gonzalez A. Vázquez-Baeza Y. Jiang L. Casals-Pascual C. . (2023). Determination of effect sizes for power analysis for microbiome studies using large microbiome databases. Genes 14, 1239. doi: 10.3390/genes14061239 37372419 PMC10297957

[B48] R Core Team (2022). R: A Language and Environment for Statistical Computing. (Vienna, Austria: R Foundation for Statistical Computing). Available online at: https://www.R-project.org/ (Accessed May 12, 2026).

[B49] Shi J. Gao G. Yu Z. Wu K. Huang Y. Wu L.-P. . (2022). The relevance of host gut microbiome signature alterations on de novo fatty acids synthesis in patients with multi-drug resistant tuberculosis. Infection. Drug Resist. 15, 5589–5600. doi: 10.2147/IDR.S372122 36168638 PMC9509681

[B50] Suzek B. E. Wang Y. Huang H. McGarvey P. B. Wu C. H. UniProt Consortium (2015). UniRef clusters: a comprehensive and scalable alternative for improving sequence similarity searches. Bioinformatics 31, 926–932. doi: 10.1093/bioinformatics/btu739 25398609 PMC4375400

[B51] Takagi T. Inoue R. Oshima A. Sakazume H. Ogawa K. Tominaga T. . (2022). Typing of the gut microbiota community in Japanese subjects. Microorganisms 10, 664. doi: 10.3390/microorganisms10030664 35336239 PMC8954045

[B52] UniProt Consortium (2019). UniProt: a worldwide hub of protein knowledge. Nucleic Acids Res. 47, D506–D515. doi: 10.1093/nar/gky1049 30395287 PMC6323992

[B53] Wang S. Yang L. Hu H. Lv L. Ji Z. Zhao Y. . (2022). Characteristic gut microbiota and metabolic changes in patients with pulmonary tuberculosis. Microb. Biotechnol. 15, 262–275. doi: 10.1111/1751-7915.13761 33599402 PMC8719804

[B54] Whittington A. M. Turner F. S. Baark F. Templeman S. Kirwan D. E. Roufosse C. . (2023). An acidic microenvironment in tuberculosis increases extracellular matrix degradation by regulating macrophage inflammatory responses. PloS Pathog. 19, e1011495. doi: 10.1371/journal.ppat.1011495 37418488 PMC10355421

[B55] Wingett S. W. Andrews S. (2018). FastQ Screen: a tool for multi-genome mapping and quality control. F1000Research 7, 1338. doi: 10.12688/f1000research.15931.2 30254741 PMC6124377

[B56] Winglee K. Eloe-Fadrosh E. Gupta S. Guo H. Fraser C. Bishai W. (2014). Aerosol Mycobacterium tuberculosis infection causes rapid loss of diversity in gut microbiota. PloS One 9, e97048. doi: 10.1371/journal.pone.0097048 24819223 PMC4018338

[B57] World Health Organization (2024). Global Tuberculosis Report 2024 (Geneva: World Health Organization).

[B58] Wu G. Zhao N. Zhang C. Lam Y. Y. Zhao L. (2021). Guild-based analysis for understanding gut microbiome in human health and diseases. Genome Med. 13, 22. doi: 10.1186/s13073-021-00840-y 33563315 PMC7874449

[B59] Ye S. Wang L. Li S. Ding Q. Wang Y. Wan X. . (2022). The correlation between dysfunctional intestinal flora and pathology feature of patients with pulmonary tuberculosis. Front. Cell. Infect. Microbiol. 12, 1090889. doi: 10.3389/fcimb.2022.1090889 36619765 PMC9811264

[B60] Yu Z. Shen X. Wang A. Hu C. Chen J. (2023). The gut microbiome: a line of defense against tuberculosis development. Front. Cell. Infect. Microbiol. 13, 1149679. doi: 10.3389/fcimb.2023.1149679 37143744 PMC10152471

[B61] Yunusbaeva M. Borodina L. Terentyeva D. Bogdanova A. Zakirova A. Bulatov S. . (2024). Excess fermentation and lactic acidosis as detrimental functions of the gut microbes in treatment-naive TB patients. Front. Cell. Infect. Microbiol. 14, 1331521. doi: 10.3389/fcimb.2024.1331521 38440790 PMC10910113

