# Supplementary material for: Bifidobacterium- and Escherichia-dominant ecological guilds shape altered microbial metabolic capacity of the gut microbiome in tuberculosis patients
Source: Front Cell Infect Microbiol. 2026 Jul 21;16:1751447. doi: 10.3389/fcimb.2026.1751447 (PMC13433188; doi:10.3389/fcimb.2026.1751447)
Supplement: Supplementary file 1 [file Table1.docx]

**Supplementary Information**

Table of Contents

Supplementary Figure 1. Empirical cutoffs for defining elevated proportions of atypical enterosignature	2

Supplementary Figure 2. Effect sizes (Cohen's d) of tuberculosis status on gut microbiome taxonomic and functional measures	3

Supplementary Table 1. Effect sizes (Cohen's d) of tuberculosis status on gut microbiome taxonomic and functional measures	4

Supplementary Figure 3. Gut microbiome diversity and composition in ТB patients and healthy controls	5

Supplementary Table 2. Clinical and demographic characteristics of the compared groups	6

Supplementary Figure 4. Taxonomic features of the gut microbiota of TB patients and controls	7

Supplementary Figure 5. Linear discriminant analysis (LDA) effect size scores for metabolic pathways enriched in ES-Esch	8

Supplementary Figure 6. Linear discriminant analysis (LDA) effect size scores for metabolic pathways enriched in ES-Bifi	9

Supplementary Figure 7. Summary of metabolic pathway types enriched in ES-Esch and ES-Bifi	10

Supplementary Figure 8. Metabolic features of the gut microbiota in the overall TB patient cohort compared to healthy controls	11

**A**

**Supplementary Figure 1. Empirical cutoffs for defining elevated proportions of atypical enterosignature.** Histograms show that most healthy controls (90%) have near-zero abundances of ES-Esch and ES-Bifi in fecal microbiomes (n = 47). The red dashed line indicates the 90th percentile point of the empirical distribution, which was chosen as the cutoff.

**Supplementary Figure 2. Effect sizes (Cohen's d) of tuberculosis status on gut microbiome taxonomic and functional measures**

Power curves were computed using a significance level of 0.05.

The first (top) row shows: (A) Power estimates based on the effect size of tuberculosis (Cohen's d = 0.368) on taxonomic diversity (Shannon index); (B, C) Power estimates based on the effect of tuberculosis on taxonomic variation captured by PC1 (d = 3.855) and PC2 (d = 0.274).

The second (bottom) row shows: (D) Power estimates based on the effect size of tuberculosis (Cohen's d=1.362) on metabolic pathway diversity (Shannon index), as inferred using HUMAnN analysis; (E, F) Power estimates based on the effect size of tuberculosis on metabolic pathway variation captured by PC1 (d = 1.336) and PC2 (d = 1.208).

**Supplementary Table 1. Effect sizes (Cohen's d) of tuberculosis status on gut microbiome taxonomic and functional measures**

| **Measure** | **Cohen's d** | **95% CI** | **p-value**** | **Interpretation*** |
| --- | --- | --- | --- | --- |
| Taxonomic diversity (Shannon species) | 0.368 | 0.12 – 0.61 | 0.004 | Small / Moderate |
| Taxonomic variation (PC1) | 3.855 | 3.12 – 4.59 | < 0.001 | Very large |
| Taxonomic variation (PC2) | 0.274 | -0.17 – 0.72 | 0.52 | Negligible (not significant) |
| Metabolic pathways diversity (Shannon pathways) | 1.362 | 0.86 – 1.86 | < 0.001 | Large |
| Metabolic pathways (PC1) | 1.336 | 0.84 – 1.83 | < 0.001 | Large |
| Metabolic pathways (PC2) | 1.208 | 0.70 – 1.72 | < 0.001 | Large |

*Interpretation based on Cohen's thresholds: 0.2 = small, 0.5 = medium, 0.8 = large. These thresholds are descriptive; cross‑outcome comparisons should be interpreted with caution;

**p-values are derived from the Cohen's d confidence intervals (CI not crossing zero corresponds to p < 0.05)

**Supplementary Figure 3. Gut microbiome diversity and composition in ТB patients and healthy controls.** (A) A boxplot illustrating the Shannon diversity index, which represents the overall taxonomic diversity in the control and TB groups. No significant differences in diversity were observed between the TB and control groups (P > 0.05). (В, С) PCA analysis based on CLR-transformed relative abundances of bacterial taxa (В) and metabolic pathways (С). PC1 and PC2 are given with the proportion of total variance explained.

**Supplementary Table 2. Clinical and demographic characteristics of the compared groups**

|  | **TB patients**  **(N =33)** | **Healthy control**  **(N =47)** | **p-value** |
| --- | --- | --- | --- |
| Gender (Male) | 18/33 (54.5%) | 24/47 (51.1%) | 0.7589 |
| Age, years (Median (IQR) | 41 (36-52.5) | 36 (29.5-40) | 0.0001 |
| Clinical forms of pulmonary tuberculosis | |  |  |
| Infiltrative TB | 19/33 (57.6%) | .. |  |
| Disseminated TB | 13/33 (39.4%) | .. |  |
| Caseous pneumonia | 1/33 (3.0%) | .. |  |

Data are median (IQR) (first and third quartiles) or n/N (%), unless stated otherwise. N ― number of individuals

**Supplementary Figure 4. Taxonomic features of the gut microbiota of TB patients and controls.**

LDA score plots were generated using the LEfSe analysis. The length of the bar column represents the LDA score. Plots show the microbial taxa with significant differences between the TB patients (red) and healthy controls (green) at the order (A), family (B), and genus (C) level (LDA score > 2.5).

**Supplementary Figure 5. Linear discriminant analysis (LDA) effect size scores for metabolic pathways enriched in ES-Esch (LDA index > 2.5) in TB patients (red) versus their respective healthy controls (green)**. Key pathways of energy metabolism are highlighted in yellow.

**Supplementary Figure 6. Supplementary Figure 5. Linear discriminant analysis (LDA) effect size scores for metabolic pathways enriched in ES-Bifi (LDA index > 2.5) in TB patients (red) versus their respective healthy controls (green)**. Key pathways of energy metabolism are highlighted in yellow.

| А |  |
| --- | --- |
| В |  |

**Supplementary Figure 7. Summary of metabolic pathway types enriched in ES-Esch (А) and ES-Bifi (В).** The block size reflects the number of unique metabolic pathways belonging to a particular type of metabolic pathway.

**Supplementary Figure 8. Metabolic features of the gut microbiota in the overall TB patient cohort compared to healthy controls (unstratified analysis).**

LDA score plots were generated using the LEfSe analysis. The length of the bar column represents the LDA score. Plots show metabolic pathways with significant differences between the TB patients (red) (LDA score > 2.5). In the gut microbiota of TB patients, the top metabolic pathways demonstrating the greatest differentiation from healthy controls are predominantly associated with ES-Esch (represented by black squares) and ES-Bifi (indicated by turquoise squares).
